# Prevalence of methotrexate intolerance in rheumatoid arthritis and psoriatic arthritis

**DOI:** 10.1186/ar4413

**Published:** 2013-12-18

**Authors:** Maja Bulatović Ćalasan, Oscar FC van den Bosch, Marjonne CW Creemers, Martijn Custers, Antonius HM Heurkens, Jan Maarten van Woerkom, Nico M Wulffraat

**Affiliations:** 1 Department of Pediatric Immunology, Room KC 03.063.0, University Medical Center, Wilhelmina Children’s Hospital, Utrecht 3508 AB, The Netherlands; 2 Department of Rheumatology, Jeroen Bosch Hospital, ‘s-Hertogenbosch, The Netherlands; 3 Department of Rheumatology, Woerden, Maartenskliniek, The Netherlands; 4 Department of Rheumatology, Meander Medical Center, Amersfoort, The Netherlands; 5 Department of Rheumatology, Gelre Hospitals, Apeldoorn and Zutphen, The Netherlands

## Abstract

**Introduction:**

The aim of this study was to determine the prevalence of gastrointestinal and behavioural symptoms occurring before (anticipatory/associative) and after methotrexate (MTX) administration, termed MTX intolerance, in rheumatoid (RA) and psoriatic arthritis (PsA).

**Methods:**

Methotrexate Intolerance Severity Score (MISS), previously validated in juvenile idiopathic arthritis patients, was used to determine MTX intolerance prevalence in 291 RA/PsA patients. The MISS consisted of four domains: abdominal pain, nausea, vomiting and behavioural symptoms, occurring upon, prior to (anticipatory) and when thinking of MTX (associative). MTX intolerance was defined as ≥6 on the MISS with ≥1 point on anticipatory and/or associative and/or behavioural items.

**Results:**

A total of 123 patients (42.3%) experienced at least one gastrointestinal adverse effect. The prevalence of MTX intolerance was 11%. MTX intolerance prevalence was higher in patients on parenteral (20.6%) than on oral MTX (6.2%) (p < 0.001).

**Conclusion:**

Besides well-known gastrointestinal symptoms after MTX, RA and PsA patients experienced these symptoms also before MTX intake. RA and PsA patients on MTX should be closely monitored with the MISS for early detection of MTX intolerance, in order to intervene timely and avoid discontinuation of an effective treatment.

## Introduction

Rheumatoid arthritis (RA) and psoriatic arthritis (PsA) are inflammatory disorders characterized by chronic arthritis [1,2]. In RA and PsA treatment, methotrexate (MTX) is the first-choice disease-modifying anti-rheumatic drug (DMARD) due to low costs, efficacy and an acceptable safety profile [3,4]. Serious adverse effects such as pulmonary toxicity, hepatotoxicity and bone marrow suppression are rare or transient if MTX is stopped [5]. In contrast, gastrointestinal adverse effects are common, affecting as many as 66% of patients [2,6-11]. Due to these adverse effects, up to 12% of RA and PsA patients discontinue MTX after 6 months to 2 years of treatment [6-8,12].

Previously, we showed in juvenile idiopathic arthritis (JIA) that 50.5% of patients suffered not only from a myriad of gastrointestinal adverse effects after MTX intake, but also from adverse effects before MTX intake (anticipatory) and when thinking of MTX (associative) [13]. The latter symptoms arise as a classical conditioning response to gastrointestinal symptoms after MTX administration. Therefore, the nature of MTX-induced gastrointestinal adverse effects, which we termed MTX intolerance, is complex, and could even further impede the use of an otherwise effective drug. Although MTX-induced gastrointestinal adverse effects occur frequently in RA and PsA, severity and the type - in particular the occurrence of anticipatory and associative symptoms - have not been assessed.

The aim of this study was to determine the type and prevalence of MTX-induced gastrointestinal adverse effects, with a standardized questionnaire, in a large cohort of RA and PsA patients.

## Methods

### Study design and patients

A cross-sectional descriptive study (ISRCTN13524271) included RA and PsA patients attending the outpatient wards of four general hospitals between May 2011 and June 2012. All patients were treated with MTX for at least 3 months and received weekly folic acid (5 to 15 mg) [5]. Patients’ data on disease activity, MTX dose and route of administration, co-medication, history of peptic ulcers and smoking was collected. The study was approved by the medical ethics committees of the University Medical Center Utrecht and the four general hospitals in ‘s-Hertogenbosch, Woerden, Amersfoort and Apeldoorn where the patients were included. As the study burden for patients was low and required no treatment changes, the ethics committees waived the need for informed consent.

### MTX intolerance severity score

To determine the prevalence of MTX-induced gastrointestinal adverse effects, patients completed the methotrexate intolerance severity score (MISS), previously developed and validated in JIA [13]. The MISS consists of four domains: abdominal pain, nausea, vomiting and behavioural symptoms, assessing symptoms after MTX administration, anticipatory (before MTX) and associative symptoms (when thinking of MTX). The behavioural symptoms domain includes restlessness, irritability and refusal of MTX, which develop in response to MTX-induced gastrointestinal symptoms and anticipation thereof. A patient could score 0 (no symptoms), 1 (mild symptoms), 2 (moderate symptoms) or 3 (severe symptoms) points on each item. MTX intolerance was defined as ≥6 points, including at least one anticipatory, associative or behavioural symptom [13].

### MTX intolerance prevalence

The prevalence was determined of: a) individual symptoms in all patients; b) MTX intolerance, defined as above; c) individual symptoms in MTX intolerant versus tolerant patients. MTX intolerance prevalence was compared between patients on oral and parenteral MTX (chi-square test). MTX intolerance severity, age, MTX dose, disease activity parameters and medication use were compared in tolerant versus intolerant patients, and in intolerant patients on oral versus parenteral MTX (*t*-test, Mann–Whitney *U*-test). To evaluate associations of MTX intolerance with clinically relevant covariates - disease activity score (DAS)-28, physician global assessment (PGA), age, MTX dose, MTX route and non-steroidal anti-inflammatory drug (NSAID) use - multivariate logistic regression was performed. Statistical analyses were carried out with IBM SPSS Statistics, version 20.

## Results

### Baseline characteristics

Of 296 patients, 5 were excluded due to diagnosis other than RA or PsA (n = 3 with ankylosing spondylitis; n = 1 with peripheral spondyloarthritis; and n = 1 with scleroderma). Table 1 shows the baseline characteristics of 291 patients; the majority was female (62.2%), 249 (85.6%) had RA and 42 (14.4%) had PsA with low to moderate DAS-28.

**Table 1:** Baseline characteristics of 291 patients at the time of completing the MISS

**Characteristic**	**Value in patient sample**
Sex, female	181 (62.2)
Age, mean +/- SD years	59.4 +/- 12.4
Diagnosis	
Rheumatoid arthritis	249 (85.6)
Psoriatic arthritis	42 (14.4)
Disease activity	
Disease activity score 28, median (IQR)^a^	2.5 (1.7 to 3.2)
Physician’s global assessment, median (IQR), (0 to 10 scale)^b^	2.0 (1.0 to 3.0)
Erythrocyte sedimentation rate (mm/hour)	11.0 (5.0 to 22.0)
MTX use	
Route of administration, oral	194 (66.7)
Dose, mg/week, median (IQR)	20.0 (12.5 to 25.0)
Other medication	
NSAIDs	145 (49.9)
Proton-pump inhibitors	127 (43.6)
Anti-emetics	5 (1.7)
Oral steroids	31 (10.7)
Other DMARDs^c^	72 (24.7)

Characteristics are as calculated at the time of completing the MISS except where indicated otherwise. Values are number (%), except where indicated otherwise. ^a^Disease activity score 28 was determined in 266 patients (274 rheumatoid arthritis and 19 psoriatic arthritis patients); ^b^physician’s global assessment was determined in 268 patients; ^c^of 72 patients on other DMARDs, 26 were on DMARDs (plaquenil, n = 24; leflunomide, n = 2) and 46 were on biologic agents (infliximab, n = 24; adalimumab, n = 10; etanercept, n = 9; abatecept, n = 2; golimumab, n = 1). MISS, methotrexate intolerance severity score; MTX, methotrexate; NSAIDs, nonsteroidal anti-inflammatory drugs; DMARDs, disease-modifying anti-rheumatic drugs.

### MTX intolerance prevalence in RA and PsA

One hundred and twenty-three (42.3%) RA and PsA patients experienced at least one gastrointestinal symptom during MTX treatment. The most prevalent gastrointestinal symptom after MTX administration was nausea, affecting 93 (32.0%) patients, whereas abdominal pain occurred in 11.3% and vomiting in 6.5% (Table 2). Pre-treatment nausea was the most prevalent; 8.6% had anticipatory and 11.0% associative nausea. Anticipatory vomiting was the least prevalent, affecting 1.7% (Table 2). Behavioural symptoms, overall, affected 16.5% of patients, with restlessness being the most prominent symptom in 13.1% of patients (Table 2).

**Table 2:** Prevalence of MTX-related gastrointestinal symptoms in all patients and in intolerant patients by route of MTX administration

	**All patients**	**Tolerant to MTX**	**Intolerant to MTX**	**Oral MTX**	**Parenteral MTX**
Total	291 (100)	259 (89.0)	32 (11.0)	194 (66.7)	97 (33.3)
Cutoff score ≥6	32 (11.0)	0 (0)	32 (100)	12 (6.2)	20 (20.6)^a^
Abdominal pain	44 (15.1)	24 (9.3)	20 (62.5)	7 (58.3)	13 (65.0)
After MTX	33 (11.3)	18 (6.9)	15 (46.9)	5 (41.7)	10 (50.0)
Anticipatory	17 (5.8)	5 (1.9)	12 (37.5)	5 (41.7)	7 (35.0)
Associative	17 (5.8)	6 (2.3)	11 (34.4)	4 (33.3)	7 (35.0)
Nausea	100 (34.4)	68 (26.3)	32 (100)	12 (100)	20 (100)
After MTX	93 (32.0)	61 (23.6)	32 (100)	12 (100)	20 (100)
Anticipatory	25 (8.6)	7 (2.7)	18 (56.3)	6 (50.0)	12 (60.0)
Associative	32 (11.0)	15 (5.8)	17 (53.1)	5 (41.7)	12 (60.0)
Vomiting	22 (7.6)	11 (4.2)	11 (34.4)	5 (41.7)	6 (30.0)
After MTX	19 (6.5)	9 (3.5)	10 (31.3)	5 (41.7)	5 (25.0)
Anticipatory	5 (1.7)	0 (0)	5 (15.6)	2 (16.7)	3 (15.0)
Behavioural symptoms	48 (16.5)	22 (8.5)	26 (81.3)	7 (58.3)	19 (95.0)^b^
Restlessness	38 (13.1)	16 (6.2)	22 (68.8)	6 (50.0)	16 (80)
Irritability	29 (10.0)	7 (2.7)	22 (68.8)	5 (41.7)	17 (85.0)^c^
Refusal of MTX	13 (4.5)	1 (0.4)	12 (37.5)	2 (16.7)	10 (50.0)

Values are number (%) of patients. All domains and individual items differentiate between tolerant and intolerant patients (*P* < 0.001). ^a^*P* < 0.001 versus oral MTX, by chi-square test; ^b^*P* = 0.02 versus oral MTX, by chi-square test, ^c^*P* = 0.02 versus oral MTX, by chi-square test. MTX, methotrexate.

MTX intolerance was found in 32 (11.0%) patients having a median score of 9 (IQR: 6.25 to 12.00). The prevalence and severity of MTX intolerance was similar in RA (n = 26 (10.4%), score 9 (6.8 to 12.3)) and PsA (n = 6 (14.3%), score 7 (6.0 to 13.0)). All intolerant patients (100%) experienced post-treatment nausea, whereas 46.9% had post-treatment abdominal pain and 31.3% had post-treatment vomiting (Table 2). The most prevalent pre-treatment gastrointestinal symptoms were anticipatory and associative nausea, affecting 56.3% and 53.1% of intolerant patients respectively, followed by anticipatory abdominal pain in 37.5% and associative abdominal pain in 34.4%. Anticipatory vomiting occurred in 15.6% of intolerant patients, whereas this symptom did not occur in tolerant patients. Overall, behavioural symptoms occurred in 81.3% of intolerant patients, of whom 37.5% refused MTX.

MTX-intolerant patients were younger than the MTX-tolerant (mean age 51.6 +/- 12.2 versus 60.4 +/- 12.1 years, *P* < 0.001). MTX dose, DAS-28, PGA, erythrocyte sedimentation rate (ESR), co-medication use, history of peptic ulcers (3.8% of all patients) and smoking (25.8% of all patients) did not differ between the two groups. Gender distribution did not differ between MTX-intolerant and MTX-tolerant patients (female, 75.0% versus 60.6%; male, 25.0% versus 39.4%), but more female (75%) than male patients (25%) were intolerant, although this was not statistically significant.

### MTX intolerance prevalence in patients on oral and parenteral MTX

MTX intolerance prevalence was significantly higher in patients on parenteral (20 of 97, 20.6%) than on oral MTX (12 of 194 m 6.2%, *P* < 0.001) (Table 2). Significantly more patients on parenteral than on oral MTX exhibited behavioural symptoms (*P* = 0.02), whereas other symptoms were comparable between the two groups. The median MTX intolerance score was higher in intolerant patients on parenteral than on oral MTX, although not significantly (9.5, IQR 7.0 to 15.5) versus 7.5, IQR 6.0 to 9.0), *P* = 0.08). Patients on parenteral MTX received the same MTX dose (20.0 mg/week, IQR 15.0 to 25.0) as patients on oral MTX (20.0 mg/week, IQR 15.0 to 20.0).

In the multivariate analysis, older patients were less likely to have MTX intolerance (odds ratio (OR) 0.93, 95%m, CI 0.89, 0.97; *P* = 0.001). If age was stratified into two groups, namely ≥65 and <65 years, older patients were again less likely to have MTX intolerance (OR 0.21, 95% CI 0.06, 0.85; *P* = 0.03) whereas patients with higher PGA (OR 1.26, 95% CI 1.05, 1.51; *P* = 0.01) and those receiving parenteral MTX (OR 3.88, 95% CI 1.41, 10.62; *P* = 0.01) were more likely to have MTX intolerance.

## Discussion

We showed that besides the well-known MTX-induced gastrointestinal symptoms upon MTX administration, RA and PsA patients also had anticipatory and associative gastrointestinal and behavioural symptoms before MTX administration, collectively termed MTX intolerance. MTX intolerance prevalence in RA and PsA patients was 11%.

Studies in RA have found similar occurrence rates compared to our study [7,11]; nausea was the most prevalent symptom, occurring in 14.4 to 28.0% compared to 32.0% in our cohort, followed by abdominal pain in 9.7 to 10.6% compared to 11.3% in our cohort and vomiting in 3.4% compared to 6.5% in our cohort. Of note is that comparisons were made between symptoms occurring only after MTX, as it is likely that previous studies took solely these symptoms into account (not the pre-treatment symptoms).

In contrast to JIA in which the prevalence of MTX intolerance reached 50.5%, the prevalence in RA/PsA was considerably lower at 11%. MTX intolerance severity was lower in adults (score 9) than in children (score 12) (*P* = 0.003). Substantially lower MTX intolerance prevalence in RA/PsA was due to: a) lower percentage of adults with score ≥6, and b) lower percentage of adults (24.4% versus 67% in JIA) with at least one anticipatory, associative and/or behavioural symptoms. As anticipatory and associative symptoms arise as classic conditioning responses to physical symptoms upon MTX use, the lower percentage of RA/PsA patients with pre-treatment symptoms suggests a weaker, classic, conditioning response in adults than in children taking MTX. This is supported by the fact that, whereas 82% of 204 JIA patients with symptoms after MTX also had symptoms before MTX intake, only 51% of 106 RA/PsA patients with symptoms after MTX had symptoms before MTX intake.

MTX intolerance prevalence was higher in patients on parenteral (20.8%) than on oral MTX (6.2%), which we also demonstrated for JIA [13,14]. This difference was caused by more behavioural symptoms in the parenteral group. Aversion towards needles, besides aversion towards MTX, could have contributed to a higher prevalence of these symptoms. It is common to switch patients from oral to parenteral MTX due to gastrointestinal symptoms [5]. Indeed, 13 of 20 intolerant patients on parental MTX had been switched to this route from oral MTX due to gastrointestinal symptoms. Considering their past symptoms on oral MTX, the patients who switched may have been more prone to develop gastrointestinal and behavioural symptoms on parenteral MTX, resulting in higher MTX intolerance prevalence in the parenteral group.

Besides the observed association between parenteral MTX and MTX intolerance, age was also associated with MTX intolerance, namely older patients (>65 years) were less likely to have MTX intolerance than younger patients (≤65 years). In previous studies, neither younger nor older age (>65 years) was associated with occurrence of MTX-related gastrointestinal and other side effects [15,16]. Validation studies are required to determine whether younger age is a risk factor for MTX intolerance.

Anticipatory and associative gastrointestinal symptoms could have a negative impact on patients’ quality of life [14] and impede the use of MTX. Nevertheless, these symptoms are clinically not very evident [13]. Consequently, they cannot be easily detected by physician assessment only, but can be detected using the MISS [13]. Therefore, using the MISS is advantageous as it allows early detection of symptoms. This could create a window of opportunity for timely treatment of MTX intolerance, as well as for early treatment of emerging physical symptoms, which could prevent the development of conditioned responses and therefore MTX intolerance. Similar to JIA, treatment of (physical) symptoms could include lowering the MTX dose [17], switching to parenteral MTX [14,18,19] or starting behavioural therapy [20] or anti-emetics [19].

Although the MISS was validated and employed to measure MTX intolerance prevalence in JIA, it provided a structured platform to assess the type of MTX-induced gastrointestinal symptoms in RA/PsA. Nevertheless, the MISS should be validated in adults with rheumatic diseases. Furthermore, this study does not reveal variables associated with MTX intolerance development, nor does it demonstrate the frequency of MTX discontinuation or of switching to other medication due to MTX intolerance. Prospective trials are required to address these issues.

This is the first study to demonstrate using a standardized questionnaire, that MTX intolerance occurs in 11%, more frequently in patients on parenteral than on oral MTX, and possibly persists after a switch from oral to parenteral MTX. Since persistent gastrointestinal symptoms are the major reason to discontinue MTX, intolerant patients could be more prone to stop MTX or switch to (less effective) DMARDs or expensive biological agents [12]. Upon validation in adults, the MISS may be used in daily clinical practice to closely monitor patients and to intervene timely using the abovementioned approaches in order to prevent or reduce the negative impact of MTX intolerance on patients’ daily lives, compliance and continuation of an effective treatment.

## Conclusions

Using a standardized MISS questionnaire, we showed that besides the well-known MTX-induced gastrointestinal symptoms upon MTX administration, RA and PsA patients also experienced anticipatory and associative gastrointestinal and behavioural symptoms before MTX administration, which develop as a classical conditioning response to physical symptoms after MTX. The prevalence of MTX intolerance was 11%. MTX intolerance occurred more often in patients on parenteral (20.6%) than in those on oral MTX (6.2%) and persisted after a switch from oral to parenteral MTX. As persisting MTX intolerance could have a negative impact on patients’ quality of life and hamper the use of MTX, RA and PsA patients on MTX should be monitored with the MISS for early detection of MTX intolerance. This would create a window of opportunity to intervene timely and avoid incompliance and discontinuation of an otherwise efficacious treatment.

## Abbreviations

DAS: Disease activity score; DMARD: Disease-modifying anti-rheumatic drug; ESR: Erythrocyte sedimentation rate; JIA: Juvenile idiopathic arthritis; MISS: Methotrexate intolerance severity score; MTX: Methotrexate; NSAID: Non-steroidal anti-inflammatory drug; OR: Odds ratio; PGA: Physician global assessment; PsA: Psoriatic arthritis; RA: Rheumatoid arthritis.

## Competing interests

The authors declare that they have no competing interests.

## Authors’ contributions

MBĆ: substantial contribution to conception and design, acquisition of data, analysis and interpretation of data; involved in drafting the manuscript and revising it critically for important intellectual content; final approval of the version to be published. OvdB: substantial contribution to acquisition of data, analysis and interpretation of data; involved in drafting the manuscript; final approval of the version to be published. MCr: substantial contribution to acquisition of data; involved in revising the manuscript critically for important intellectual content; final approval of the version to be published. MCu: substantial contribution to acquisition of data; involved in revising the manuscript critically for important intellectual content; final approval of the version to be published. AH: substantial contribution to acquisition of data; involved in revising the manuscript critically for important intellectual content; final approval of the version to be published. JMvW: substantial contribution to acquisition of data; involved in revising the manuscript critically for important intellectual content; final approval of the version to be published. NW: substantial contribution to conception and design and interpretation of data; involved in revising the manuscript critically for important intellectual content; final approval of the version to be published. All authors read and approved the final manuscript.
